# On the Treatment of Rheumatic Fever

**Published:** 1864-05

**Authors:** Willoughby F. Wade

**Affiliations:** Senior Physician to the Queen’s Hospital, Birmingham, England


### SELECTED.

### ON THE TREATMENT OF RHEUMATIC FEVER.

By WILLOUGHBY F. WADE, M. D., M. R., C. P

Senior Physician to the Queen's Hospital, Birmingham.

[*Concluded.*]

So great is the constitutional irritation, that rheumatic fever has been treated exclusively by opium, and this in large quantities, for the disorder establishes an extraordinary tolerance of the drug. Dr. Corrigan, of Dublin, is the present supporter of the plan. I have myself given it a very fair trial, but I cannot acquiesce in his faith in its efficiency. It is true that it tranquilizes and solaces the sufferer, and in many cases rest and diluents may during this time effect a cure; but on the other hand, I have seen patients lying for days semi-narcotized, the rheumatic fever itself undiminished. It may, nevertheless, be well-conjoined with other more efficacious methods, when the patient is unusually irritable, restless, or incapable of enduring pain, whilst these are working a cure. At the same time I rarely find it necessary to use it; and, as a rule, it is desirable to simplify, and not to complicate, treatment or multiply drugs, else it becomes difficult to distinguish their effects.

The treatment so far indicated does but pave the way for that which is to exercise a speedy and unerring influence upon the course and duration of the disorder. As soon as the patient has taken the powder above directed, he is to commence the use of the following:—Nitrate of potash, ʒj; acetate of potash, ʒiij; water, ℥viij; ℥j for a dose. This is to be repeated every two, three, or four hours, according to the urgency of the symptoms. At the same time the patient is to be kept upon slops, and supplied with copious libations of barley, toast, or plain water, thin gruel, weak broths, and so on.

The result of this treatment is, that the urine is increased in quantity and becomes less acid, the nervous irritation is soothed, the heart's action quieted, the arthritic effusion diminished, the pains are allayed; and all this in from twenty-four to seventy-two hours. The patient is often so much relieved by the first night, that he is pleased and comforted by the improvement, and is content without any anodyne, lying quiet and composed, though very likely unable to obtain much or at all events uninterrupted sleep.

Other saline remedies will, I believe, produce similar effects. Thus, Dr. Todd, recommends bicarbonate and nitrate of potash; Dr. Weber, bicarbonate of soda; Dr. Garrod, bicarbonate of potash; Dr. Fuller, a mixture of several salts. Lemon juice alone is employed by Dr. Owen Rees, who believes that its oxygen is directed to those tissues, the *im*perfect oxydation of which develops the rheumatic poison. Be that what it may lemon juice is often efficacious; but my experience of it coincides with that of those who have found it uncertain, and therefore unreliable. I have been so satisfied with the results obtained from the use of the nitrate and acetate of potash since I first employed them, that I have rarely tried any similar drug. The only objection to this mixture is that it sometimes gripes, an unpleasantness which may always be corrected by increasing the quantity of water in which it is administered, or adding to it a little compound tincture of cardamoms. The remarks above made regarding lemon-juice apply also to colchicum, which has the further disadvantage of being oftentimes violent and distressing in its action.

It is quite true that steady persistence in the use of whichever we may select of these salines will be followed in from twenty-four to seventy two hours by a very considerable alleviation of the symptoms, both local and general; and further that this amelioration will sometimes progress till a perfect cure ensues. But it is a peculiarity of rheumatic which does not pertain to most other idiopathic fevers, that there is constant tendency to relapse, and this relapse occurs either before the cure is apparently complete, or even after that period. A rheumatic fever of six weeks' duration is not one and indivisible like a typhoid or typhus fever, but rather a succession of rheumatic fevers linked together by periods of partial recovery—i. e., it is a series of relapses; if not of relapses, of migrations, one joint getting well while another becomes inflamed. This *erratic* character of rheumatism is most prone to manifest itself in persons who are anæmiated or debilitated from any cause. It may be that it simply wanders from joint to joint, one not getting well till another is invaded; or it may happen that all the joints get well for a period; or again it may happen that all the joints get partially well for a period, and then occurs a relapse. Again, it may happen that whereas for a day or two the patient makes a steady progress towards recovery, yet after that time the disorder comes to a halt. The remedies which for a time seemed to be acting so well, cease to exert any influence. This, I say, will occur under any of the methods which may be classed together under Dr. Todd's group of "eliminative plans."

To show the frequency of these relapses, I quote from several recent authors who have recorded cases for various purposes but none of them avowedly with the intention of exemplifying this particular point. Of 10 cases related by Dr. Bennett ("Principles and Practice of Medicine"), 3 had relapses. Out of 5 cases mentioned by Dr. Todd, 2 had relapses, and 1 lingered on without change for two weeks. Out of 6 cases narrated by Dr. Fuller, 2 relapsed. Out of 23 cases recorded by Dr. Whitley, which were treated by various physicians of Guy's Hospital, 9 had relapses (one of them two); and a 10th case seems to have had one, but the record is not explicit.

Dr. Basham records 65 cases of acute arthritic rheumatism, and of these 9 had relapses, some of them having had several. Judging from other circumstances, I should think it probable that these nine had become almost if not quite convalescent before that attack which was termed a relapse; if so, it is not unfair to assume that many more had relapses before recovering pretty completely from the first invasion.

Of course the secondary attacks varied much in severity, but they present instances of pericarditis, pleurisy, and irritis, either constituting or attending the relapse, and then occurring for the first time.

It is unnecessary to multiply proofs of the tendency to relapse which I have alleged to be characteristic of this complaint, since of 109 cases 25 relapsed—some several times. Here, then, is a feature which demands our most earnest attention, since its pernicious tendencies are bu inefficiently opposed by any recognized plan of treatment. How are we to meet this difficulty?

Rheumatism, whether acute or chronic, like its congener, gout, is more prone to wander in persons whose constitutions are enfeebled; and, as I have already pointed out, many of these patients have suffered from anæmia from a date long antecedent to their arthritic affection. Here, then, seems a plain indication for treatment: support and corroborants—meat, wine, steel, quinine, bark, and so on. The first case which strongly enforced this view upon my mind was that of a girl whom I had charge of at the General Hospital some years ago. It was one of those cases in which the symptoms, though not *very* severe, were yet persistent, although migratory. She was evidently anæmic. One day, she asked for a glass of wine, and I gave it to her; the next day she was better, and I continued to give her two ounces of wine daily, and from that time she steadily convalesced.

Iron is a "strengthening" remedy, but as a rule it is not well borne by a pyrexial system, and it commonly checks secretion, so that it must be used cautiously in the rheumatic habit. When I was in Paris in 1851, M. Briquet, of the Charité, was engaged in making experiments and observations upon the administration of quinine in acute cases of this fever. He had not finished them when I left; but I had seen quite enough to convince me that it was no sheet-anchor when given from the first in all cases, and I had also seen enough to convince me that cases so treated would some of them get well almost by magic. A much older physician than M. Briquet —Dr. Haygarth, a man of celebrity in his time—was in the habit of treating all his cases from the first with bark. Here is an important fact. It is quite true, as Dr. Fuller points out, that published records of his cases indicate that bark is not a safe remedy when thus used; still the fact remains that Dr. Haygarth treated 121 cases with bark (more or less completely), and was at the end a most strenuous advocate of the plan.

When, then, my attention was first directed to the necessity of preventing relapses, quinine presented itself to my mind as promising to be an efficient agent.

Dr. Fuller uses quinine earlier than most other persons, and the few cases he has published seem to my mind indicative of its utility. His remarks upon it are as follows:—"In my own practice bark has never been given at such an early (from the third to the tenth day) period of the disease, nor have I often seen it so administered by others; but I have repeatedly watched its administration at a later period, while the tongue has still continued furred, and the pulse excited; and it has been so constantly followed by a fresh accession of mischief, that I have been deterred from making use of it until the urine has cleared or has dropped its sediment, the pulse has become soft, and the tongue moist and almost clean." Of quinine he says, it "is more readily and earlier tolerated; and as it is quite as efficient as bark, it should certainly have the preference when the *eruption of sudamina*, the character of the pulse, or the *cleaning of the tongue*, appear to demand or admit of the exhibition of a tonic. It should be used as a corrective or restorative of the processes of assimilation when the febrile paroxysm is beginning to abate, rather than as a cure during the active stages of the disease."

If, however, we wait till the tongue is quite clean, or the urine normal, and the fever gone, or sudamina are developed, we shall wait in many cases till a relapse, affecting perhaps the pericardium or pleura, has occurred. Nor can we tell beforehand, with anything like certainty, in what cases this will or will not happen.

The plan which I have adopted for some time, then, has been to administer from two to four grains of quinine every three or four hours, *as soon as ever there is a distinct remission in the symptoms:* this happens, as I have said, in from twenty-four to seventy-two hours after the commencement of the treatment. At this period, however, the patient is far from convalescent. The joints, probably, are still painful and swollen, though less so than at first; the pulse perhaps is still quicker than natural, yet it has come down 10 or 15 beats; the tongue is moist and cleaner, though still perhaps loaded with fur.

But whilst I use the quinine in this way to prevent a relapse which occurs more or less decidedly in 25 out of 109 cases treated in the ordinary way, I cannot trust to it to eliminate the residue of the rheumatic poison in the body, nor to eliminate that small quantity which continues to be formed whilst the quinine is producing its effect, which latter seems to me to be antagonistic to the production of the rheumatic poison. I therefore, whilst giving the quinine every four hours, continue the use of the potash mixture night and morning; and I give of it one or two ounces, according to the fever, pain, and swelling still existing. On the same day that I begin the quinine or on the next, I allow the patient some meat, and, if he be evidently feeble, a glass or two of wine. The meat I do not press upon him, but if he desires to have it he has it.

When the rheumatism is quite gone, I generally employ steel, and usually in the form of iodide of iron, the iodine acting gently on the excretory organs, whilst the iron helps to restore the blood-globules, so often, as we have seen, deficient. If one or more joints seem to progress less rapidly than the others, it is useful to apply a small blister, as recommended by Dr. Todd; and during convalescence, if any remain, as they often do, stiff and perhaps a little swollen, I paint them with that application common in most hospitals under the name of pigmentum iodinii. Having employed this plan for some years in both public and private practice, I can strongly recommend its adoption, and the more confidently since it has proved as efficacious in the hands of others as in my own.

